# Photoinactivation of Staphylococci with 405 nm Light in a Trachea Model with Saliva Substitute at 37 °C

**DOI:** 10.3390/healthcare9030310

**Published:** 2021-03-11

**Authors:** Tobias Meurle, Johannes Knaus, Agustin Barbano, Katharina Hoenes, Barbara Spellerberg, Martin Hessling

**Affiliations:** 1 Institute of Medical Engineering and Mechatronics, Ulm University of Applied Sciences, 89081 Ulm, Germany; jknaus@mail.hs-ulm.de (J.K.); barbano@mail.hs-ulm.de (A.B.); katharina.hoenes@thu.de (K.H.); 2 Institute of Medical Microbiology and Hygiene, University of Ulm, 89081 Ulm, Germany; Barbara.Spellerberg@uniklinik-ulm.de

**Keywords:** ventilator-associated pneumonia, visible light photoinactivation, endotracheal tube, ESKAPE pathogens, 405 nm, artificial human saliva, trachea replacement model, prevention of VAP

## Abstract

The globally observed rise in bacterial resistance against antibiotics has increased the need for alternatives to antibiotic treatments. The most prominent and important pathogen bacteria are the ESKAPE pathogens, which include among others *Staphylococcus aureus*, *Klebsiella pneumoniae* and *Acinetobacter baumannii*. These species cause ventilator-associated pneumonia (VAP), which accounts for 24% of all nosocomial infections. In this study we tested the efficacy of photoinactivation with 405 nm violet light under conditions comparable to an intubated patient with artificial saliva for bacterial suspension at 37 °C. A technical trachea model was developed to investigate the visible light photoinactivation of *Staphylococcus carnosus* as a non-pathogen surrogate of the ESKAPE pathogen *S. aureus* (MRSA). The violet light was coupled into the tube with a fiber optic setup. The performed tests proved, that photoinactivation at 37 °C is more effective with a reduction of almost 3 log levels (99.8%) compared to 25 °C with a reduction of 1.2 log levels. The substitution of phosphate buffered saline (PBS) by artificial saliva solution slightly increased the efficiency during the experimental course. The increased efficiency might be caused by a less favorable environment for bacteria due to for example the ionic composition.

## 1. Introduction

In the last years increased concern was voiced about the spread of a range of antibiotic-resistant bacteria, known as the ESKAPE pathogens, which are responsible for most nosocomial infections [1,2]. One of the most problematic nosocomial infections are ventilator-associated pneumonia (VAP), provoked by intubation for mechanical ventilation.

A study from 2020 demonstrated an incidence of VAP of 4.6 to 5.4 cases per 1000 ventilation days, depending on the surveillance system [3]. In a meta-analysis Melsen et al. [4] examined 24 clinical trials concerning VAP, estimating the mortality of the infection to be at 13%. Mechanical ventilation leads to impairment of the patient’s natural protective mechanisms, while microaspiration at the cuff of the tube [5,6] favors proliferation of pathogens to the lung, causing pneumonias. Formation of biofilm on the endotracheal tube (ETT) is suggested to complicate VAP prevention by acting as bacterial reservoir, which is difficult to eradicate [7,8,9,10]. VAP is one of the most common nosocomial infections in intensive care units, which not only leads to prolonged hospital stays and higher treatment costs [3,11] but is also associated with a high mortality [4,12].

The increase in antibiotic resistance and the reduction of research for new antibiotics pose a challenge to health care systems, which strongly rely on antibiotics for the successful treatment of bacterial infections [13,14,15,16,17]. Therefore, alternative treatment and prevention approaches are urgently needed [18,19].

One promising alternative is the photoinactivation with visible light, which has been tested for different applications aiming at a reduction of microbial organisms [20,21,22,23]. While UV-radiation causes damage to the deoxyribonucleic acid (DNA) of human cells [24], visible light is more suitable for less toxic applications on humans [23]. Multiple studies have demonstrated that violet and blue light are especially effective for photoinactivation applications [20,25,26].

In the pursuit of reducing the risk of VAP, Sicks et al. [27] integrated 450 nm LEDs in a conventional ETT and achieved a reduction of *Staphylococcus carnosus* by three log levels. These successful experiments were performed in phosphate buffered saline (PBS) at room temperature, conditions which differ considerably from the situation within a patient and may thus influence the results. Therefore, a more realistic imitation of the clinical situation was developed, that includes a temperature of 37 °C and a different choice of storage medium mimicking subglottic secretions. In this improved trachea model, which can be heated to 37 °C, we substituted PBS by an artificial saliva salt solution.

Additionally, an optical fiber approach was tested, which could have advantages compared to LEDs incorporated in an ETT, because it does not require an electrical current within the human body and the related risk considerations that might complicate the medical approval procedure. An optical fiber could also be combined with existing ETTs of any size and without the need to develop and produce new ETTs.

This setup can be employed to illuminate the improved trachea model with arbitrary wavelengths and intensities and was used here to investigate the antimicrobial effect of 405 nm (violet) light at room temperature (25 °C) and human body temperature (37 °C) on *S. carnosus* as *Staphylococcus aureus* surrogate.

## 2. Materials and Methods

### 2.1. Microorganism and Medium

*S. aureus* and *Staphylococcus epidermis* together account for 36% of hospital-acquired respiratory infections [17], but due to regulations for the available laboratory, which do not allow culturing bacterial pathogens, the test organism for the disinfection experiments was its non-pathogenic relative *S. carnosus* (DSM no. 20501). This strain was obtained from Deutsche Sammlung von Mikroorganismen und Zellkulturen GmbH (Braunschweig, Germany) and cultivated in a tryptic soy yeast extract medium (M92). The growth medium consisted of 30 g tryptic soy broth, 3 g yeast extract and 1000 g distilled water. The pH was adjusted to 7.0–7.2. Prior to irradiation, *S. carnosus* was cultivated in this growth medium for 4 h at 37 °C on a rotary shaker at 170 rpm. In the next step, the medium was removed and the resultant pellet was washed in PBS. Resuspended cells were diluted in the specific solution for the certain irradiation experiment until a starting concentration of about 2 × 10^7^ colony forming units (CFU) per mL was reached for experimental use.

### 2.2. Illuminated Endotracheal Tube Prototype

A high-power light emitting diode (LED) LEUVA66 × 00VV00 from LG (Seoul, Korea) was chosen for the investigation. The optical power of this LED was 2200 mW at its peak wavelength 405 nm and its radiation angle amounted to 120°. The LED had no built-in lens and therefore had a planar surface, which is especially important for the subsequent fiber optic cable coupling. The polymer fiber optic cable with a laterally emitting segment on the lower part, was obtained from Ilumae GmbH (Dresden, Germany) with a diameter of 2.1 mm, a total length of 680 mm and a laterally radiation area of 80 mm. After coupling the LED in the fiber optic cable, an integrating sphere setup revealed a homogeneous irradiance with an average of 3.57 mW/cm^2^ at the laterally radiating surface of the fiber optic cable.

### 2.3. Test Setup 

**Figure 1:**
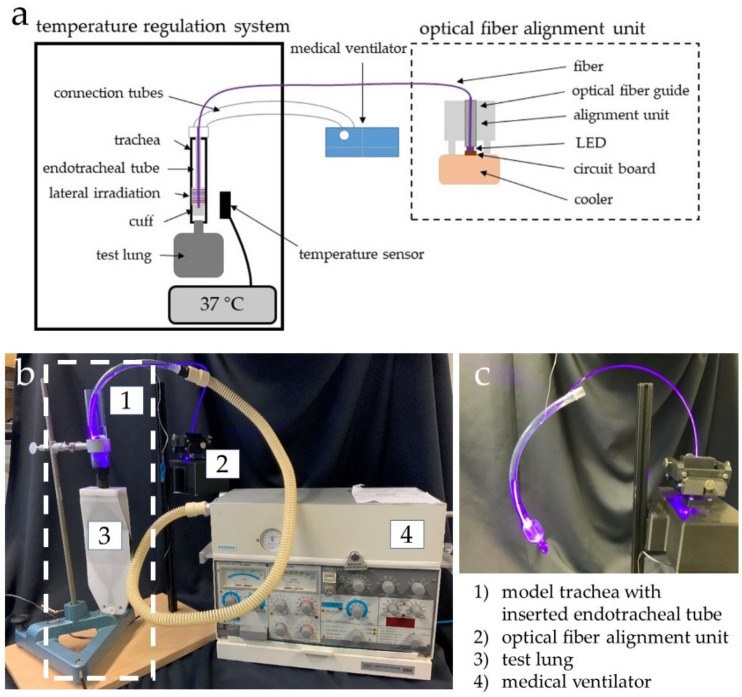
Schematic test setup of temperature regulated trachea model with LED based fiber optic integrated in an ETT and the lateral bacterial irradiation (**a**), photograph of the test setup with the dashed box indicating the 37 °C tempered part (**b**) and endotracheal tube with inserted optical fiber emitting 405 nm light in the area around and above the cuff (**c**).

Figure 1 illustrates the test setup with its components, described in the following. The fiber was inserted in an ETT, so that the laterally emitting area of the fiber irradiated the area above the cuff. The Endosid^®^-ETT had an inner diameter of 10 mm and was produced by Asid Bonz GmbH (Herrenberg, Germany). The ETT was connected to a medical ventilator ELMA AB Servo Ventilator SV 900 C from Siemens (Erlangen, Germany), which generated an airflow of 7 L/min in the ETT, as it would have been expected for a ventilated patient. A trachea model with an adjustable temperature was designed, which consists of an enclosed heated system with a temperature controller. In the model, a glass tube replaced the trachea. The LED-based fiber optic ETT was inserted into the glass tube and sealed at the bottom by pressurizing the cuff. In addition, a non-transparent conical tube was positioned close to the irradiated sample for the control sample. To achieve the maximum irradiation the fiber position relative to the LED could be adjusted in two directions in the optical fiber alignment unit.

### 2.4. Microbiological Experiments

**Figure 2:**
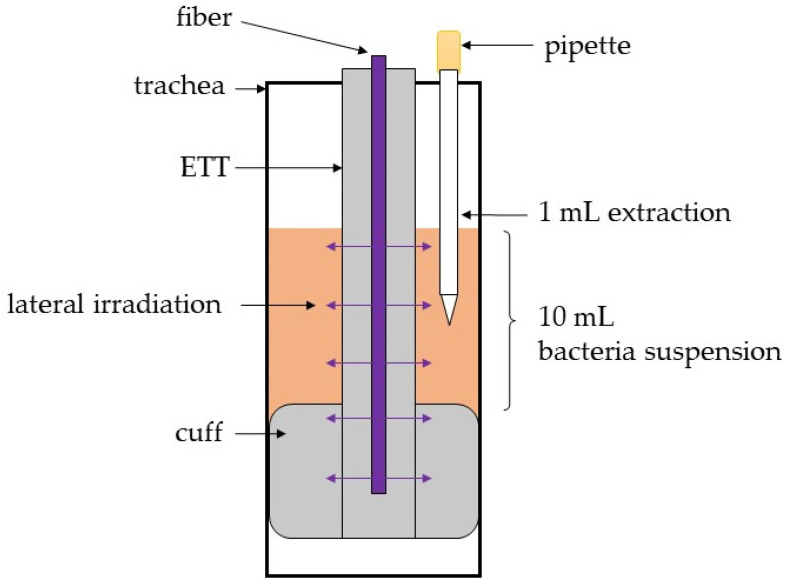
Scheme of sample drawing process in the trachea model.

The space above the cuff—between ETT and glass tube—was filled with the bacterial test solution, as described in Figure 2. In each experiment, 10 mL of the *S. carnosus* solution were filled into the trachea model and in the control vessel, respectively. The samples were taken at the predetermined times 0, 2, 3, 4, 5, 7 and 9 h. To achieve more realistic patient conditions, the system temperature was set to body temperature of 37 °C. 

For the determination of CFU in irradiated and control samples 33.3 µL of samples were cultured on M92 agar plates in different dilutions. Each sample was plated in triplicates and each individual test run was repeated three times on separate days. After incubation at 37 °C for 24 h, the colonies were counted manually and converted in CFU/mL.

#### 2.4.1. *S. carnosus* Irradiation in PBS at 37 °C and 25 °C

To investigate the effect of different temperatures on the photoinactivation, the experiments in PBS were conducted in triplicates at 25 °C and 37 °C. The temperature of 25 °C was chosen for better comparability with literature results, as most studies were conducted at room temperature, while 37 °C represents human body temperature.

#### 2.4.2. Artificial Saliva Solution Comparison at 37 °C

According to Feldman et al. [28], contamination of the upper airways occurs in a first step, and circulation subsequently enables colonization of the lower airways. To mimic upper airway conditions, artificial saliva solutions were prepared and tested on *S. carnosus*. 

Candidates, which were already examined in different applications as a substitute for saliva were preferred. Therefore the collection provided by Pytko-Poloncyk et al. [29] was consulted as a reference. The provided list was examined for the ingredients of provided saliva solutions concerning possible antimicrobial effects or if there are known interactions with photoinactivation, which could possibly falsify the results. The saliva substitute options not including the composition or not providing concentrations of the ingredients were excluded [30,31,32]. The solution suggested by Gal et al. [33,34] could not be replicated in our lab environment since we do not have the facilities for CO_2_ gassing. Likewise, the substitute option provided by Arvidson et al. [35] had to be excluded due to lack of required equipment. The artificial saliva used by Alshali et al. [36] was discarded due to the high concentration of Na CMC which is not a part of real saliva [37] and therefore not to be expected in such high concentrations in the trachea of an intubated patient. From the saliva substitutes provided by Pytko-Poloncyk et al. [29] multiple candidates had to be eliminated [33,34,38,39,40,41] since they are containing potassium thiocyanate (KSCN), which has been proven by St Denis et al. [42] to have a potentiating effect on photodynamic therapy.

Additionally, other possibilities for saliva substitutes were also considered like mucin-based artificial media [43,44,45], similar to commercially available saliva substitutes, which can be applied to treat xerostomia [46]. Due to the antimicrobial and growth-inhibiting properties of mucin, no mucin-based candidate was selected for testing [47,48,49]. Another candidate was a real human saliva based approach as developed by de Jong et al. [50], but there has been concern about the substitute containing lysozyme, which is known to be an antimicrobial agent. Furthermore, a growth medium was chosen for testing. The employed medium was described by Pratten et al. [51] and was applied to grow biofilms of *Streptococcus sanguis*, an oral microorganism. Even though growth in this medium was expected, it was tested as to determine if the increase in bacterial concentration was pronounced within the given time and conditions.

The previously described selection of possible candidates led to four applicable artificial saliva solutions, with their compositions listed in Table 1. Samples of bacteria exposed to those artificial saliva replacement solutions were processed and evaluated after 9 h without irradiation at 37 °C according to the colony quantification method. A duration of 9 h was chosen because this was the predetermined duration of the irradiation experiments. 

Selection of the most adequate saliva substitution solution for irradiation experiments was determined by evaluating the antimicrobial properties or the capability of supporting bacterial growth. Both should not occur and the saliva substitute revealing the smallest occurrence of growth or reduction was chosen for further investigation. The growth and reduction were determined by comparing the bacterial concentrations in the different saliva substitutes to the behavior in PBS, which served as control.

#### 2.4.3. *S. carnosus* Irradiation in Sal3 at 37 °C

After evaluation of the best choice of an artificial saliva substitute for the prototype testing, it was of interest to investigate the effect of the saliva solution on the photoinactivation effect of *S. carnosus*. Irradiation experiments with a duration of 9 h were conducted in Sal3 at 37 °C and then compared to the results of the PBS experiments at 37 °C to determine a potential influence of the new environment.

### 2.5. Statistical Analysis

The data of the irradiation experiments are represented by the averages of three independent experiments with triplicate samples. The data was analyzed using the R software for statistical computing in the release version 4.0.0. The data was tested using a two-sample t-Test with a significant difference being accepted at *p* < 0.05 for the sample against the corresponding control and *p* < 0.1 when comparing different parameter combinations.

## 3. Results

**Figure 3:**
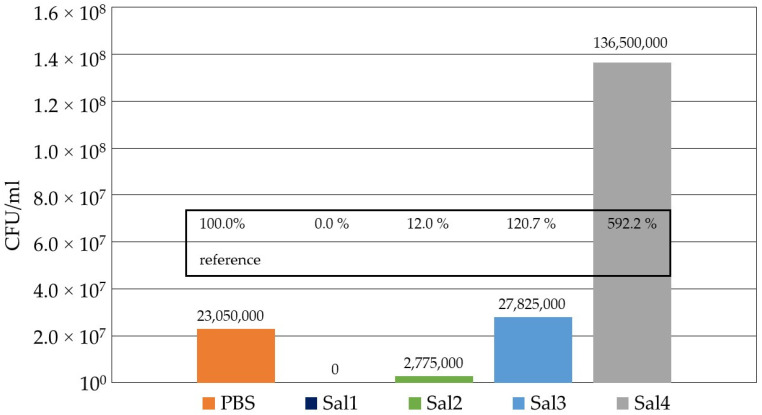
Comparison of the influence of the four selected artificial saliva substitutes on *S. carnosus* after 9 h at 37 °C compared to the control with *S. carnosus* in PBS.

The evaluation of the four artificial saliva substitutes compared to PBS is depicted in Figure 3 for an incubation time of 9 h at 37 °C without irradiation. The CFU concentration in PBS after 9 h incubation was set to 100%, whereas lower values indicate reduction and higher values display growth of the bacterial population within the incubation period, respectively. The experiment revealed that Sal1 is not suitable as a saliva substitute for photoinactivation prototype testing, as all bacteria have been eradicated just by the solution’s influence prior to any irradiation approach. Both Sal2 with only 12% remaining bacterial concentration and Sal4, which supported bacterial growth considerably and produced a bacterial concentration almost 6 times higher than in PBS, were rejected as a substitute. With a 20% higher CFU concentration compared to PBS, indicating moderate bacterial growth, Sal3 proved to be a suitable saliva substitute and was applied for further irradiation experiments.

**Figure 4:**
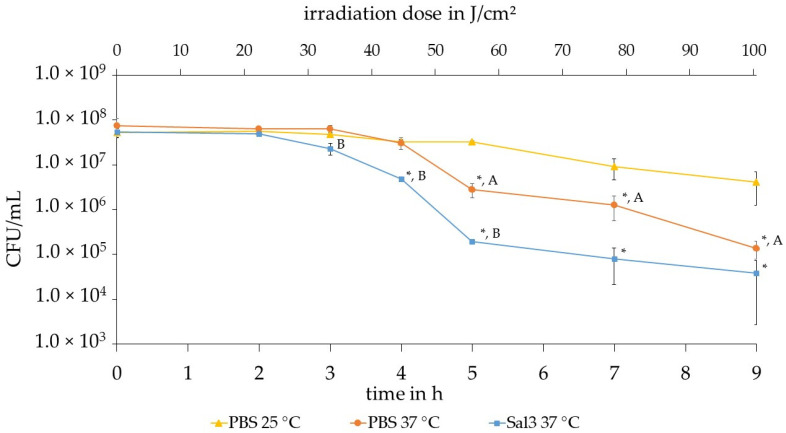
Comparison of the results of experiments with 405 nm irradiated *S. carnosus* at different temperatures and in different solutions. The curves represent triangle: 25 °C in PBS, circle: 37 °C in PBS and square: 37 °C in Sal3 with the mean deviation as errors. * represents a significant difference to the corresponding control, A represents a significant difference between PBS 25 °C and PBS 37 °C and B represents a significant difference between PBS 37 °C and Sal3 37 °C.

The results of the three irradiation experiments with different parameter combinations are displayed in Figure 4. Each experiment provides the mean value of the counted *S. carnosus* colonies of the irradiated bacterial suspension in the trachea model over the sample collection times 0 h, 2 h, 3 h, 4 h, 5 h, 7 h and 9 h. The data is presented with the mean deviation and the achieved reduction of the bacterial concentration in logarithmic scale. In all three experiments depicted in Figure 4 the 405 nm irradiation achieves a successful photoinactivation of the bacterial concentration compared to the control samples.

The maximum reduction effect is reached after 9 h at a dose of 100 J/cm^2^ with a 405 nm irradiation. It can be observed that in PBS at 25 °C, a bacterial reduction of 1.17 log (93.24%) is achieved. At a temperature of 37 °C in PBS, a reduction of 2.80 log (99.84%) is recorded. The artificial saliva salt solution (Sal3) at a temperature of 37 °C exhibits a reduction of 3.02 log (99.90%). 

A shoulder effect is visible in all three experiments, but at different times and for different radiation doses. A shoulder effect is the occurrence of an initial reduction delay resulting in an increase in the efficiency of the irradiation with increasing time. In the case of PBS at 25 °C, a shoulder effect is visible for the first 5 h. Analyzing the results at a temperature of 37 °C the shoulder effect can be detected until 4 h at an irradiation exposure of 45 J/cm^2^. In the saliva substitute Sal3, a shoulder effect is also observed, but only for the first 2 to 3 h, which corresponds to an irradiation of approximately 25–35 J/cm^2^. It is noticeable that especially in the period of 2 to 5 h a significant reduction of *S. carnosus* occurred.

It can be observed in Figure 4 that in PBS at 25 °C bacterial reduction of 1.17 log (93.24%) is achieved whereas at a temperature of 37 °C in the same environment a higher reduction of 2.80 log (99.84%) is recorded. Subsequent from 4 h the two temperatures display a statistically significant difference, when tested with a two-sample student’s test with a significance level of α = 0.1. The higher temperature is more effective starting from 4 h than at 25 °C. The irradiation in artificial saliva salt solution (Sal3) exhibits an even higher reduction of 3.02 log (99.90%) at a temperature of 37 °C.

While the PBS experiments at 25 °C and 37 °C continue to exhibit a similarly flat course and hardly any bacterial reduction up to 4 h, a bacterial reduction by 1.0 log is already visible for Sal3 at 37 °C at 4 h. From hour 4 to 5, irradiation at the lower temperature of 25 °C continues to show only a slight reduction. In this interval, the graphs of Sal3 at 37 °C and PBS at 37 °C display the same slope. Irradiation in Sal3 at 37 °C however already achieves a bacterial reduction of about 2.1 log levels at an irradiation dose of about 56 J/cm^2^, while in PBS at 37 °C only a reduction of 1.0 log level is achieved. After 5 h, the PBS experiment at 37 °C reveals a steeper descent than the other two experiments until the end of the experiment to achieve a reduction of 2.8 log levels, which is a similar result as for Sal3 at 37 °C.

## 4. Discussion

In recent years, microbial eradication by photoinactivation with visible light shifted into focus due to an increasing demand for new disinfection techniques. While the general effectiveness of photoinactivation with the light source being integrated into an ETT has been demonstrated before by Sicks et al. [27] the setup was not tested under conditions mimicking ventilation conditions. Therefore, the aim of this study was to create a test setup to determine how the alteration of study parameters could influence photoinactivation results. 

Since the development of VAP is one of the most important clinical issues related to mechanical ventilation [3], different attempts for its prevention have been tested before. The presented approach and the one by Hashemi et al. [55] both modified ETTs to inhibit the growth of microbial pathogens. While in our study visible light was added to achieve this, Hashemi et al. [55] added an antimicrobial coating to an ETT. On the other hand there are also attempts to reduce the occurrence of VAP by changing the treatment of ventilated patients, like oral disinfection [56,57], subglottic drainage [58,59,60] and cuff pressure control [61,62].

While these attempts may improve the outcome, they always require the staff to increase their care of the ventilated patients or add steps to the daily routine and are therefore prone to human errors.

Similar fiber setups, as in this study, have been employed by Huang et al. [63] and Shehatou et al. [64] for other applications. Huang et al. [63] have designed their setup to reduce the risk of catheter-associated urinary tract infections (CAUTI). Their applied light setup was, similar to the one presented here, composed of a laser diode and a diffusing fiber. However, there are some important differences between the study presented by Huang et al. and this one, as they employed red light with a wavelength of 660 nm and a fluence rate of 50 mW/cm^2^ in a female rat model. Furthermore, they applied external photosensitizers, which makes the inactivation results incomparable to those of our study. 

The setup and methods applied by Shehatou et al. [64] exhibit more similarities to the one used in this study. Both employ 405 nm light for an in vitro study with a light delivery system, which is based on an optical fiber. The irradiation experiments of Shehatou et al. [64] were performed with ESKAPE pathogens since the goal of that study was to reduce the risk of nosocomial infections. While they proved that the inactivation of all ESKAPE pathogens is possible, they also tested the inactivation of *S. aureus* in mice lung surfactant. They achieved a 4 log level reduction of *S. aureus* after 250.8 J/cm^2^ in MH medium, human serum and lung surfactant at room temperature. While the results at the highest applied dose were comparable for different media, the dose-dependent photoinactivation progress was different. The occurrence of an inactivation effect was first observed in medium, then in human serum and latest in lung surfactant. Similar results were achieved in this study, where the overall results after 9 h irradiation was comparable for PBS and saliva solution, but the progress was different and the reduction slope more pronounced in saliva solution.

Their experiment in comparison with literature data about the irradiation resistance of lung epithelia cells lead Shehatou et al. [64] to the conclusion, that their approach might not be applicable to the treatment of lung infections. However, since the purpose of the integration of light sources into an ETT in our study is not to treat lung infections, but to inactivate bacteria in the trachea before they reach the lung and therefore prevent infections, the conclusion of Shehatou et al. [64] is not applicable for this study.

In Table 2 different approaches applying visible light with the intention to profit from photoinactivation effects by implementation inside the human body are summarized.

The present investigation was performed with *S. carnosus* instead of *S. aureus*, raising the question about the comparability of the two species. Hoenes et al. [71] have previously compared the *S. carnosus* strain used in this study, with a *S. aureus* strain and discovered that the photoinactivation of *S. aureus* is not only possible with the same conditions but even requires a smaller dose for 1 log level reduction. Photoinactivation has also been demonstrated to be effective against *S. aureus* by others using different setups [26,72].

The results obtained in this study at 37 °C in PBS match well to the results achieved on *S. carnosus* by Hoenes et al. [71]. While 1.88 log levels were reduced in the trachea model with an irradiance of 3.57 mW/cm^2^ emitted from the optical fiber, a reduction in bacterial concentration of 1.56 log levels was reached in the laboratory setting of Hoenes et al. with a homogenizing optical element for the single 405 nm LED and an irradiance of 20 mW/cm^2^. Apparently, differences in irradiance do not play a major role in photoinactivation with 405 nm as long as a sufficient dose is applied. This phenomenon was also observed before by other researchers [22,23,73,74]. 

The increased potential for bacterial reduction at a higher temperature compared to room temperature, which we similarly demonstrated here for 25 °C and 37 °C in PBS (Figure 4), was also observed in the study by McKenzie et al. [75]. Their study compared the photoinactivation effects for *E. coli* and *Listeria monocytogenes* at 4, 22 and 45 °C. While McKenzie et al. compared different temperatures that differ from the ones tested in our study, photoinactivation at 45 °C also exhibited an increased effect for both bacterial species in comparison to room temperature.

In this study additionally, the impact of an artificial saliva at body temperature of 37 °C on the photoinactivation results was investigated. For this proceeding first of all suitable saliva substitutes had to be identified. Sal3 was chosen as the most appropriate substitute for further investigations concerning photoinactivation, since there occurred no inherent antimicrobial effect.

Comparison of PBS and Sal3 at the same temperature demonstrated, that Sal3 provides a slightly more inactivation-promoting environment than PBS. While this is not clearly distinct for the last measuring point at 9 h, which shows a similar bacterial reduction for both, it is visible throughout the earlier time points, including the shoulder effect. This is illustrated in Figure 4, where bacteria in PBS at 37 °C develop the shoulder effect until 4 h of irradiation, while in Sal3 this occurs only up to 2 to 3 h.

A possible explanation for this difference compared to PBS, is that the bacterial cells are already in an environment, which weakens the cell capabilities to withstand or repair the damages caused to the cells by photoinactivation in Sal3, like the ionic composition. McKenzie et al. [75] demonstrated before that the salt concentration in the surrounding medium has an impact on the inactivation results of *E. coli* and *L. monocytogenes* with 405 nm light. This is backed by Rath et al. [76] who determined, that altering the salinity has an impact on microbial processes in soil. This could cause the required dose for the photoinactivation effect to be decreased.

## 5. Conclusions

The newly added parameters in this study, namely photoinactivation in artificial saliva salt solution and at increased temperature of 37 °C, provide further evidence that photoinactivation might be a helpful approach concerning improvements of mechanical ventilation circumstances. A significant reduction of *S. carnosus* by 3 log levels (99.9%) could be demonstrated and would be conceivable as an approach for the prevention of VAP to reduce unnecessary health problems and mortality.

## Figures and Tables

**Table 1:** Composition of the four different artificial saliva replacement solutions.

Sal1 [52]	Sal2 [40,53]	Sal3 [54]	Sal4 [51]
19 mg/L MgCl_2_ 6H_2_0 2240 mg/L KCl 103 mg/L CaCl_2_ 544 mg/L KH_2_PO_2_	400 mg/L NaCl 400 mg/L KCl 795 mg/L CaCl_2_ 2H_2_O 690 mg/L NaH_2_PO_2_ H_2_O 5 mg/L Na_2_S 9H_2_O 1000 mg/L Urea (CH_4_N_2_O)	4200 mg/L NaHCO_3_ 500 mg/L NaCl 200 mg/L KCl	1 g meat extract 2 g yeast extract 5 g proteose peptone 0.2 g NaCl 0.2 g KCl 0.3 g CaCl_2_ 1.25 mL Urea 40% sterile filtered 1000 mL aqua dem.

**Table 2:** Visible light-based approaches for intended application inside the human body.

Target Organ	Aim	Application	Reference
trachea	prevention of VAP	integration of 48 miniature LEDs in wall of ETT to reduce bacterial concentration in accumulating secretion at cuff, in vitro model, 450 nm, 6.6/13.4 mW/cm^2^, 280/480 J/cm^2^, room temperature	[27]
trachea	prevention of biofilm formation on ETT	in vitro polymicrobial biofilm of *P. aeruginosa* and *S. aureus*, light delivery by catheter, methylene blue (500 µg/mL) + 664 nm, 150 J/cm length of catheter	[65]
stomach	therapy of *H. pylori* colonization and correspondent diseases, especially in cases of antibiotic failure	in vivo, 10 patients with symptoms of dyspepsia or suspected peptide ulcer disease and tested positive for *H. pylori*, optical fiber passed through endoscope, 405 nm, 40 J/cm^2^	[66]
stomach	therapy of *H. pylori* infection	in vivo, 18 patients, fiber optic bundle with diffusor with over-tube for flow of coolant to maintain 45° C, catheter-sheath in form of a multi-segmented balloon for positioning, 408 nm, 31–46 kJ, repopulation after irradiation	[67]
stomach	therapy of gastric infections	presentation of the idea of an ingestible LED capsule, in vitro tests on *H. pylori* and prototype design, 405 nm, 460 nm, (500 nm, 625 nm)	[68,69,70]
not specified	presenting a non-traditional approach for the prevention and/or therapeutic intervention of hospital acquired infections	in vitro tests on ESKAPE-pathogens and eukaryotic cells, experiments on agar, in liquid culture (incl. lung surfactant, human serum) and on surfaces, flexible Corning^®^ light-diffusing fiber from silica glass, 230 µm diameter, 405 nm, 36–540 J/cm^2^	[64]
urinary tract	therapy of catheter-associated urinary tract infections (CAUTI)	female rat model, light delivery by direct introduction of diffusing fiber into the bladder, 660 nm, 50 mW/cm^2^, 100 J/cm^2^, external addition of photosensitizer methylene blue and salt potassium iodide prior to irradiation by catheter	[63]

## Data Availability

The data presented in this study are available on request from the corresponding author.
